# Lack of Association Between *CAT* rs1001179 and *SOD2* rs4880 Variants and Risk for Multiple Sclerosis

**DOI:** 10.3390/biom16101449

**Published:** 2026-10-04

**Authors:** Elena García-Martín, Javier Gómez-Tabales, José A. G. Agúndez, Julián Benito-León, Jorge Millán-Pascual, María Díaz-Sánchez, Patricia Calleja, Laura Turpín-Fenoll, Hortensia Alonso-Navarro, Esteban García-Albea, Félix Javier Jiménez-Jiménez

**Affiliations:** 1 Institute of Molecular Pathology Biomarkers, University of Extremadura, Av/de la Universidad S/N, 10071 Cáceres, Spain; elenag@unex.es (E.G.-M.); jagomezta@unex.es (J.G.-T.); jagundez@unex.es (J.A.G.A.); 2 CIBERNED (Centro de Investigación Biomédica en Red de Enfermedades Neurodegenerativas), Instituto de Salud Carlos III, 28029 Madrid, Spain; julian.benito@salud.madrid.org; 3 Service of Neurology, Hospital Universitario Doce de Octubre, 28041 Madrid, Spain; maria.diaz.sanchez@gmail.com (M.D.-S.); patricia.calleja@salud.madrid.org (P.C.); 4 Section of Neurology, Hospital La Mancha-Centro, Ciudad Real, 13600 Alcázar de San Juan, Spain; jorge.millan.pascual@gmail.com (J.M.-P.); lturdoc@hotmail.com (L.T.-F.); 5 Section of Neurology, Hospital Universitario del Sureste, Arganda del Rey, 28500 Madrid, Spain; hortalon@yahoo.es; 6 Department of Medicine-Neurology, Hospital “Príncipe de Asturias”, Universidad de Alcalá, Alcalá de Henares, 28801 Madrid, Spain; egarciaalbea@gmail.com

**Keywords:** multiple sclerosis, genetics, genetic polymorphisms, *SOD2* gene, *CAT* gene, risk factors

## Abstract

**Objectives:** Several lines of evidence point to a potential involvement of oxidative stress in the pathophysiology of multiple sclerosis (MS). Superoxide dismutase isoenzymes 1 and 2 (SOD1 and SOD2) and catalase (CAT) are key components of the enzymatic antioxidant defense system. Consequently, genetic variation in the genes encoding these enzymes may influence susceptibility to MS. However, previous studies examining the relationship between *SOD1*, *SOD2*, and *CAT* variants and MS susceptibility have yielded inconsistent findings. The present study aimed to investigate whether two common single-nucleotide variants (SNVs) in *CAT* and *SOD2* are associated with MS risk in a Caucasian Spanish population. **Methods:** We investigated the *CAT* rs1001179 and *SOD2* rs4880 variants in 300 patients with MS and 400 healthy controls using allele-specific TaqMan quantitative PCR assays. We further examined whether these variants were related to relevant clinical characteristics, including age at disease onset, disease severity, and clinical course/subtype. **Results:** Neither the genotype nor allele distributions of *CAT* rs1001179 and *SOD2* rs4880 differed significantly between patients with MS and healthy controls. Furthermore, no significant relationships were observed between either variant and sex, age at disease onset, disease severity, or clinical MS subtype. **Conclusions:** The present study did not detect significant associations between the *CAT* rs1001179 or *SOD2* rs4880 variants and MS susceptibility in the Caucasian Spanish population. Similarly, no significant associations were detected between these variants and the clinical characteristics evaluated. However, smaller genetic effects cannot be excluded.

## 1. Introduction

Multiple sclerosis (MS) is a chronic immune-mediated disease of the central nervous system (CNS) characterized by inflammatory processes, demyelination, and progressive neuronal damage. Genetic susceptibility is an important determinant of MS risk, and genome-wide association studies (GWAS) have identified more than 200 susceptibility loci to date [1,2]. Nevertheless, the effect sizes associated with most of these variants are relatively modest, and collectively they account for only a proportion of the genetic component of MS susceptibility [1,2]. The strongest genetic association has consistently been observed within the *HLA* region, particularly the *HLA-DRB1*15:01* haplotype [1].

Genetic susceptibility alone, however, is unlikely to fully account for the development and clinical course of MS. Several environmental exposures and interactions between genetic and environmental factors have been proposed as contributors to disease onset and progression. These include reduced sunlight exposure and consequent low vitamin D status, infectious agents—most notably Epstein–Barr virus—along with obesity and cigarette smoking [3,4,5]. Increasing evidence also points to oxidative stress as a relevant component of MS pathophysiology. Because oxidative imbalance is closely interconnected with inflammatory mechanisms [6,7,8], excessive production of reactive oxygen species and impaired antioxidant defenses may contribute to the tissue injury and neurodegenerative processes observed in MS [9,10,11,12].

Superoxide dismutase isoenzymes 1 and 2 (SOD1 and SOD2) and catalase (CAT) are among the principal enzymatic systems involved in the cellular response to oxidative stress. However, studies evaluating SOD and CAT activity in patients with multiple sclerosis (MS) have yielded inconsistent findings [12]. Increased SOD1 and SOD2 enzymatic activity has been reported in actively demyelinating lesions [13], as well as in the cerebellar gray matter of individuals with MS [14]. Reduced SOD activity has been described in erythrocytes [15,16], leukocytes [16], and peripheral blood mononuclear cells (PBMCs) [17] from patients with MS. In serum or plasma, SOD activity has been reported to be either comparable to that of controls [18,19,20], elevated [21,22,23,24], or reduced [25,26]. CAT activity has also shown substantial variability across biological compartments. Increased CAT activity has been detected in active demyelinating lesions [14], whereas erythrocyte CAT activity has been reported to be lower [27] or similar [28] in patients with MS compared with controls. CAT activity in leukocytes was comparable between groups [28], while increased activity has been observed in PBMCs from patients with MS [26]. Similarly, serum/plasma CAT activity has been described as either higher [19,21,24] or unchanged [18,28] in MS patients relative to controls.

Given the established involvement of SOD and CAT in the regulation of oxidative stress, together with the potential contribution of oxidative imbalance to MS pathophysiology, genetic variation in the genes encoding these antioxidant enzymes may hypothetically represent a potential determinant of MS susceptibility. In this context, a study involving 140 patients with MS and 142 controls reported an association between the *SOD2* rs4880 and *CAT* rs7943316 variants and altered susceptibility to MS [29]. In contrast, a more recent study comprising 200 patients with MS and 265 controls found no significant association between MS risk and either *SOD2* rs4880 or *CAT* rs1001179, although both variants were associated with selected motor and sensory manifestations and with the presence of spinal cord lesions [30]. The present study evaluated whether the *SOD2* rs4880 and *CAT* rs1001179 variants are associated with susceptibility to multiple sclerosis (MS) in an independent and larger cohort of Spanish Caucasian patients and healthy controls.

## 2. Patients and Methods

### 2.1. Patients and Controls

The study comprised 300 Caucasian Spanish patients with MS who fulfilled the McDonald criteria for definite MS [31] and had no previous diagnosis of other autoimmune or neurological disorders. The control group consisted of 400 healthy, age- and sex-matched individuals who were unrelated to the patients. Controls were eligible provided that none of their first-degree relatives had a history of autoimmune or neurological disease. The demographic and clinical characteristics of both groups are presented in Table 1.

Patients with MS were recruited from several Spanish centers: the Multiple Sclerosis Association of Madrid (N= 165), Hospital La Mancha-Centro (Alcázar de San Juan, Ciudad Real, Spain; N = 65), University Hospital “Doce de Octubre” (Madrid; N = 30), University Hospital “Príncipe de Asturias” (Alcalá de Henares, Madrid; N = 25), and University Hospital del Sureste (Arganda del Rey, Madrid; N = 15). Healthy controls were recruited at the University Hospital of Badajoz (Badajoz, Spain).

Some participants in the present cohort had previously been included in genetic association studies conducted by our group (the previously published papers resulting from these studies have been mentioned in the Supplementary Material). The oldest DNA samples had been stored for approximately 30 years at −80 °C. Before inclusion, DNA quality and genotyping performance were verified, and only samples producing reliable genotype calls were analyzed. There was no overlap between participants included in the present study and those included in previous studies analyzing the *CAT* rs1001179 and *SOD2* rs4880 variants.

### 2.2. Ethical Aspects

The study was conducted in accordance with the ethical principles established by the Declaration of Helsinki (1975) and received approval from the respective Clinical Research Ethics Committees of the University Hospital “Príncipe de Asturias” (University of Alcalá, Alcalá de Henares, Madrid, Spain; approval code PI02/2005, 27 April 2005) and the University Hospital of Badajoz (Badajoz, Spain no approval code; 1 April 2004). All participants provided written informed consent after receiving detailed information about the study procedures and objectives.

### 2.3. Genotyping of CAT rs1001179 and SOD2 rs4880 Variants

Genomic DNA was isolated from peripheral blood leukocytes obtained from venous blood samples collected from patients with MS and healthy controls. Genotyping was carried out using real-time PCR on an Applied Biosystems 7500 qPCR system with custom TaqMan assays (Life Technologies, Alcobendas, Madrid, Spain). Two common single-nucleotide variants (SNVs), *CAT* rs1001179 and *SOD2* rs4880 were selected for analysis because both are functionally relevant variants previously investigated in case–control studies of MS [29,30]. Their selection was further supported by evidence linking these variants to other neurodegenerative diseases, whereas comparable evidence for other *CAT* or *SOD2* variants remains limited. *CAT* rs1001179 is an upstream variant located on chromosome 11, whereas *SOD2* rs4880 is a missense variant located on chromosome 6 that results in a valine-to-alanine substitution at amino acid position 16 (Val16Ala) in the SOD2 protein. The corresponding TaqMan assays were C__11468118_10 and C___8709053_10, respectively.

Genotyping quality control included no-template negative controls in each run, which showed no amplification. In addition, 10% of randomly selected samples were genotyped in triplicate, with 100% concordance among determinations. The initial genotype call rates were 95% for *CAT* rs1001179 and 98% for *SOD2* rs4880. Samples with an initially unsuccessful or ambiguous result were re-genotyped, and a definitive genotype was obtained for all 700 participants. Thus, the final call rate was 100% for both variants, and no samples were excluded from the analysis because of genotyping failure.

### 2.4. Statistical Analysis

Statistical analyses were performed using SPSS Statistics version 27.0 for Windows (SPSS Inc., Chicago, IL, USA). Hardy–Weinberg equilibrium was evaluated independently in the MS and control groups using the online Hardy–Weinberg equilibrium calculator (https://codingace.net/biology/hardy_weinberg.html; accessed on 15 August 2026). Allelic and genotypic distributions were analyzed with PLINK software version 1.9 (beta 7.11) [32]. Differences in genotype and allele distributions between groups were evaluated using the chi-square test or Fisher’s exact test, as appropriate. Analyses were performed for the overall study population and separately after stratification by sex. Analyses stratified by sex, age at onset, Expanded Disability Status Scale (EDSS), and clinical subtype were considered exploratory because the study was not specifically powered to detect associations within these subgroups. For these exploratory subgroup analyses, age at onset and EDSS were dichotomized using their respective median values in the study population, 31 years and 3, to obtain groups of approximately equal size. These data-driven thresholds were not prespecified clinical cutoffs. Odds ratios and their 95% confidence intervals were calculated to estimate the magnitude and precision of the associations. To account for multiple testing, *p*-values were adjusted using the false discovery rate (FDR) method [33]. FDR correction was performed separately for genotype and allele analyses within each analysis or stratification: all genotype comparisons were included in one correction set, and all allele comparisons were included in a separate correction set. FDR-adjusted *p* values are reported as Pc. Genotype and allele frequencies were additionally compared among MS patient subgroups defined according to age at disease onset, disease severity as measured by the EDSS, and clinical disease course. The chi-square or Fisher’s exact test was used for these subgroup analyses, as appropriate.

Sample size and statistical power were estimated under a genetic model based on the frequency of the less common allele and an assumed OR of 1.5, with a two-sided α level of 0.05. The minor allele frequencies observed among healthy controls were used for these calculations. Given the sample size of the present study, the estimated statistical power was 90.3% for *CAT* rs1001179 and 96.2% for *SOD2* rs4880.

## 3. Results

After adjustment for multiple comparisons, neither genotype nor allele distributions for *CAT* rs1001179 and *SOD2* rs4880 differed significantly between patients with MS and controls. The Hardy–Weinberg equilibrium test showed no significant deviation for either polymorphism. For *CAT* rs1001179, the Hardy–Weinberg *p* values in patients and controls were *p* = 0.8222 and *p* = 0.128, respectively. For *SOD2* rs4880, the corresponding *p* values were *p* = 0.443 and *p* = 0.908, respectively. These findings remained unchanged when the analysis was performed in the overall cohort (Table 2) or separately in men and women (Table 3).

When patients with MS were divided according to age at disease onset, using the median age of 31 years as the threshold, the genotype and allele distributions of the two SNVs examined were comparable between patients with an onset at ≥31 years and those with an onset at ≤30 years (Table 4). Likewise, no significant differences in genotype or allele frequencies were observed after stratifying patients according to the median EDSS score of 3, with comparable distributions in those with EDSS < 3 and EDSS ≥ 3 (Table 5).

We further explored whether age at disease onset was related to disability, as assessed by the EDSS. Linear regression analysis revealed no significant association between these two variables (*p* = 0.610). Similarly, chronological age was not significantly correlated with EDSS scores (*p* = 0.624).

Finally, genotype and allele frequencies for *CAT* rs1001179 and *SOD2* rs4880 were broadly comparable across the three clinical courses of MS—relapsing–remitting, secondary progressive, and primary progressive—and the control group (Table 6).

## 4. Discussion

Although accumulating evidence supports a potential involvement of oxidative stress in MS pathophysiology [9,10,11,12], and altered SOD1, SOD2, and CAT activities have been documented in several tissues [13,14,15,16,17,18,19,20,21,22,23,24,25,26,27,28], the extent to which genetic variation in *SOD1*, *SOD2*, and *CAT* influences susceptibility to MS remains uncertain. This issue has received limited attention, and the available genetic association studies have yielded inconsistent findings. For example, the *SOD2* rs4880 polymorphism was associated with MS susceptibility in one study [29], whereas no significant association was detected in another [30]. Similarly, an association between the *CAT* rs7943316 variant and MS was reported by one group [29], while a different study found no association between the *CAT* rs1001179 variant and MS risk [30].

In addition to the studies conducted in MS, the *CAT* rs1001179 variant has been investigated in several neurodegenerative diseases, including Alzheimer’s disease [34,35], amyotrophic lateral sclerosis [36], and spinal muscular atrophy [37], as well as in the risk of developing motor complications associated with dopaminergic treatment in Parkinson’s disease [38,39]. Similarly, *SOD2* rs4880 is a widely investigated functional variant that has been studied in several neurodegenerative diseases, particularly Alzheimer’s disease [40,41,42], amyotrophic lateral sclerosis [36], spinal muscular atrophy [37], Parkinson’s disease [43,44,45], and motor complications of levodopa therapy in patients with Parkinson’s disease [38,39].

In this Spanish cohort, comprising 300 patients with MS and 400 controls, we did not detect a significant association between either the *CAT* rs1001179 or *SOD2* rs4880 variant and susceptibility to MS. This lack of association was observed in the overall study population and remained unchanged when the analyses were performed separately in men and women, in line with the findings previously reported by Siokas et al. [30]. Furthermore, no significant associations were detected between either of the two SNVs and age at disease onset, disability as measured by the EDSS, or the clinical course of MS.

Wigner et al. [29] reported associations of *SOD2* and CAT variants with MS risk, whereas Siokas et al. [30] found no association of *SOD2* or *CAT* variants with disease susceptibility, although both variants were associated with selected clinical manifestations and spinal cord lesions. Our susceptibility findings agree with those of Siokas et al. [30] but differ from the association reported for *SOD2* rs4880 by Wigner et al. [29]. Differences in population ancestry, sample size, clinical characteristics, and study design may contribute to these discrepancies. Additional *CAT* variants, including rs769214, rs769217 and rs7943316, warrant investigation in larger, independent cohorts. Future studies should assess a broader set of variants and incorporate prospective clinical follow-up to determine whether antioxidant-related genetic variation influences MS susceptibility or progression.

Several limitations should be considered when interpreting our findings. First, although the sample size provided adequate statistical power to detect effects of OR ≥ 1.5, the study had less power to identify smaller effects. Second, heterogeneity in disease severity may have reduced our ability to identify associations between the investigated variants and MS-related disability. Third, the analysis was restricted to two SNVs, one in each of the *CAT* and *SOD2* genes, and therefore does not exclude a contribution from other variants in these genes. Finally, the subgroup analyses were exploratory and had limited statistical power, particularly for smaller subgroups such as patients with primary progressive MS. Dichotomization of age at onset and EDSS may also have resulted in loss of information and statistical power. Therefore, the absence of statistically significant findings, particularly in the subgroup analyses, should not be interpreted as definitive evidence that smaller associations are absent.

Future research would benefit from larger prospective studies enrolling individuals shortly after MS diagnosis, with systematic genotyping and prolonged clinical follow-up. Such studies could clarify whether antioxidant-related genetic variants influence subsequent disease progression or clinical phenotype.

## 5. Conclusions

The present study did not detect significant associations between the *CAT* rs1001179 or *SOD2* rs4880 variants and susceptibility to multiple sclerosis in the Spanish Caucasian population studied. Similarly, no significant associations were detected between these variants and sex, age at disease onset, disability, or clinical course. However, smaller genetic effects cannot be excluded, and the subgroup analyses should be considered exploratory. Further studies involving larger independent cohorts and a broader range of genetic variants are warranted.

## Figures and Tables

**Table 1:** Demographic and clinical data of the series studied.

Group	MS Patients (N = 300)	Controls (N = 400)
**Age, y, mean (SD)**	43.9 (11.4)	44.1 (11.1)
**Age at onset, y, mean (SD)**	32.8 (18.2)	NA
**Female %**	207 (69.0%)	276 (69.0%)
**EDSS**	3.27 (2.44)	NA
**MS evolutive type** Relapsing-remitting MS Secondary progressive MS Primary progressive MS	162 (54.0%) 95 (31.7%) 43 (14.3%)	NA NA NA

EDSS: expanded disability status scale. NA: not applicable.

**Table 2:** Genotypes and allelic variants of *CAT* rs1001179 and *SOD2* rs4880 of patients with MS and healthy volunteers. The values in each cell represent: number (percentage; 95% confidence intervals).

	MS Patients (N = 300, 600 Alleles)	Controls (N = 400, 800 Alleles)	Intergroup Comparison OR (95% CI), *p*; Pc
**GENOTYPES**			
rs1001179 C/C	185 (61.7; 56.2–67.2)	257 (64.3; 59.6–68.9)	0.90 (0.66–1.22); 0.484; 0.581
rs1001179 C/T	102 (34.0; 28.6–39.4)	121 (30.3; 25.7–34.8)	1.19 (0.86–1.64); 0.292; 0.581
rs1001179 T/T	13 (4.3; 2.0–6.6)	22 (5.5; 3.3–7.7)	0.77 (0.39–1.57); 0.484; 0.581
rs4880 G/G	62 (20.7; 16.1–25.2)	105 (26.3; 21.9–30.6)	0.73 (0.51–1.05); 0.087; 0.522
rs4880 G/A	155 (51.7; 46.0–57.3)	201 (50.3; 45.4–55.1)	1.06 (0.78–1.43); 0.711; 0.711
rs4880 A/A	83 (27.7; 22.6–32.7)	94 (23.5; 19.3–27.7)	1.25 (0.88–1.75); 0.210; 0.581
**ALLELES**			
rs1001179 C	472 (78.7; 75.4–81.9)	635 (79.4; 76.6–82.2)	0.96 (0.74–1.24); 0.747; 0.747
rs1001179 T	128 (21.3; 18.1–24.6)	165 (20.6; 17.8–23.4)	1.04 (0.81–1.35); 0.747; 0.747
rs4880 G	279 (46.5; 42.5–50.5)	411 (51.4; 47.9–54.8)	0.82 (0.67–1.02); 0.071; 0.142
rs4880 A	321 (53.5; 49.5–57.5)	389 (48.6; 45.2–52.1)	1.22 (0.98–1.50); 0.071; 0.142

*p*, unadjusted *p* value; Pc, FDR-adjusted *p* value. FDR correction was applied separately to the complete sets of genotype and allele comparisons within each analysis.

**Table 3:** Genotypes and allelic variants of *CAT* rs1001179 and *SOD2* rs4880 in patients with MS and healthy volunteers stratified by sex. The values in each cell represent: number (percentage; 95% confidence intervals).

	MS Women (N = 207, 414 Alleles)	Control Women (N = 276, 552 Alleles)	Intergroup Comparison OR (95% CI), *p*; Pc	MS Men (N = 93, 186 Alleles)	Control Men (N = 124, 248 Alleles)	Intergroup Comparison OR (95% CI), *p*; Pc
**GENOTYPES**						
rs1001179 C/C	124 (59.9; 53.2–66.6)	178 (64.5; 58.8–70.1)	0.82 (0.57–1.19); 0.303; 0.479	61 (65.6; 55.9–75.2)	79 (63.7; 55.2–72.2)	1.09 (0.62–1.91); 0.775; 0.930
rs1001179 C/T	73 (35.3; 28.8–41.8)	83 (30.1; 24.7–35.5)	1.27 (0.86–1.86); 0.228; 0.479	29 (31.2; 21.8–40.6)	38 (30.6; 22.5–38.8)	1.03 (0.57–1.83); 0.993; 0.993
rs1001179 T/T	10 (4.8; 1.9–7.8)	15 (5.4; 2.8–8.1)	0.88 (0.39–2.01); 0.767; 0.920	3 (3.2; −0.4–6.8)	7 (5.6; 1.6–9.7)	0.56 (0.14–2.22); 0.401; 0.876
rs4880 G/G	45 (21.7; 16.1–27.4)	72 (26.1; 20.9–31.3)	0.79 (0.58–1.21); 0.270; 0.479	17 (18.3; 10.4–26.1)	33 (26.6; 18.8–34.4)	0.62 (0.32–1.19); 0.150; 0.876
rs4880 G/A	105 (50.7; 43.9–57.5)	139 (50.4; 44.5–56.3)	1.02 (0.71–1.46); 0.937; 0.937	50 (53.8; 43.6–63.9)	62 (50.0; 41.2–58.8)	1.16 (0.68–2.00); 0.584; 0.876
rs4880 A/A	57 (27.5; 21.5–33.6)	65 (23.6; 18.5–28.6)	1.23 (0.82–1.86); 0.319; 0.479	26 (28.0; 18.8–37.1)	29 (23.4; 15.9–30.8)	1.27 (0.69–2.35); 0.445; 0.876
**ALLELES**						
rs1001179 C	321 (77.5; 73.5–81.6)	439 (79.5; 76.2–82.9)	0.89 (0.65–1.21); 0.455; 0.455)	151 (81.2; 75.6–86.8)	196 (79.0; 74.0–84.1)	1.14 (0.71–1.85); 0.580; 0.580
rs1001179 T	93 (22.5; 18.4–26.5)	113 (20.5; 17.1–23.8)	1.13 (0.83–1.53); 0.455; 0.455	35 (18.8; 13.2–24.4)	52 (21.0; 15.9–26.0)	0.87 (0.54–1.41); 0.580; 0.580
rs4880 G	195 (47.1; 42.3–51.9)	283 (51.3; 47.1–55.4)	0.85 (0.66–1.09); 0.200; 0.400	84 (45.2; 38.0–52.3)	128 (51.6; 45.4–57.8)	0.77 (0.53–1.13); 0.184; 0.368
rs4880 A	219 (52.9; 48.1–57.7)	269 (48.7; 44.6–52.9)	1.18 (0.92–1.53); 0.200; 0.400	102 (54.8; 47.7–62.0)	120 (48.4; 42.2–54.6)	1.30 (0.89–1.90); 0.184; 0.368

*p*, unadjusted *p* value; Pc, FDR-adjusted *p* value. FDR correction was applied separately to the complete sets of genotype and allele comparisons within each analysis.

**Table 4:** Genotypes and allelic variants of *CAT* rs1001179 and *SOD2* rs4880 in patients with MS according to age at onset. The values in each cell represent: number (percentage; 95% confidence intervals).

	Age at Onset ≥ 31 (N = 154, 308 Alleles)	Age at Onset ≤ 30 (N = 146, 292 Alleles)	Intergroup Comparison OR (95% CI), *p*; Pc
**GENOTYPES**			
rs1001179 C/C	98 (63.6; 56.0–71.2)	87 (59.6; 51.6–67.5)	1.19 (0.75–1.89); 0.472; 0.741
rs1001179 C/T	51 (33.1; 25.7–40.6)	51 (34.9; 27.2–42.7)	0.92 (0.57–1.49); 0.741; 0.741
rs1001179 T/T	5 (3.2; 0.4–6.0)	8 (5.5; 1.8–9.2)	0.58 (0.19–1.81); 0.343; 0.741
rs4880 G/G	29 (18.8; 12.7–25.0)	33 (22.6; 15.8–29.4)	0.79 (0.45–1.39); 0.421; 0.741
rs4880 G/A	81 (52.6; 44.7–60.5)	74 (50.7; 42.6–58.8)	1.08 (0.69–1.70); 0.741; 0.741
rs4880 A/A	44 (28.6; 21.4–35.7)	39 (26.7; 19.5–33.9)	1.10 (0.66–1.82); 0.719; 0.741
**ALLELES**			
rs1001179 C	247 (80.2; 75.7–84.6)	225 (77.1; 72.2–81.9)	1.20 (0.82–1.78); 0.348; 0.490
rs1001179 T	61 (19.8; 15.4–24.3)	67 (22.9; 18.1–27.8)	0.83 (0.56–1.23); 0.348; 0.490
rs4880 G	139 (45.1; 39.6–50.7)	140 (47.9; 42.2–53.7)	0.89 (0.65–1.23); 0.490; 0.490
rs4880 A	169 (54.9; 49.3–60.4)	152 (52.1; 46.3–57.8)	1.12 (0.81–1.54); 0.490; 0.490

*p*, unadjusted *p* value; Pc, FDR-adjusted *p* value. FDR correction was applied separately to the complete sets of genotype and allele comparisons within each analysis.

**Table 5:** Genotypes and allelic variants of *CAT* rs1001179 and *SOD2* rs4880 in patients with MS according to EDSS scores. The values in each cell represent: number (percentage; 95% confidence intervals).

	EDSS Score < 3 (N = 149, 298 Alleles)	EDSS Score ≥ 3 (N = 151, 302 Alleles)	Intergroup Comparison OR (95% CI), *p*; Pc
**GENOTYPES**			
rs1001179 C/C	93 (62.4; 54.6–70.2)	92 (60.9; 53.1–68.7)	1.07 (0.67–1.70); 0.791; 0.821
rs1001179 C/T	48 (32.2; 24.7–39.7)	54 (35.8; 28.1–43.4)	0.81 (0.50–1.30); 0.375; 0.821
rs1001179 T/T	8 (5.4; 1.7–9.0)	5 (3.3; 0.5–6.2)	1.66 (0.53–5.19); 0.382; 0.821
rs4880 G/G	30 (20.1; 13.7–26.6)	32 (21.2; 14.7–27.7)	0.94 (0.54–1.64); 0.821; 0.821
rs4880 G/A	76 (51.0; 43.0–59.0)	79 (52.3; 44.4–60.3)	0.95 (0.60–1.49); 0.821; 0.821
rs4880 A/A	43 (28.9; 21.6–36.1)	40 (26.5; 19.5–33.5)	1.13 (0.68–1.87); 0.647; 0.821
**ALLELES**			
rs1001179 C	234 (78.5; 73.9–83.2)	238 (78.8; 74.2–83.4)	0.98 (0.67–1.45); 0.932; 0.932
rs1001179 T	64 (21.5; 16.8–26.1)	64 (21.2; 16.6–25.8)	1.02 (0.69–1.50); 0.932; 0.932
rs4880 G	136 (45.6; 40.0–51.3)	143 (47.4; 41.7–53.0)	0.93 (0.68–1.29); 0.674; 0.932
rs4880 A	162 (54.4; 48.7–60.0)	159 (52.6; 47.0–58.3)	1.07 (0.78–1.48); 0.674; 0.932

*p*, unadjusted *p* value; Pc, FDR-adjusted *p* value. FDR correction was applied separately to the complete sets of genotype and allele comparisons within each analysis.

**Table 6:** Genotypes and allelic variants of *CAT* rs1001179 and *SOD2* rs4880 in patients with MS according to clinical subtype of MS. The values in each cell represent: number (percentage; 95% confidence intervals).

	Relapsing-Remitting N = 162, 324 Alleles	Secondary Progressive N = 95, 190 Alleles	Primary Progressive N = 43, 86 Alleles	Intergroup Comparison Values (Secondary Progressive vs. Primary Progressive) OR (95% CI), *p*; Pc	Intergroup Comparison Values (Relapsing-Remitting vs. Primary Progressive) OR (95% CI), *p*; Pc
**GENOTYPES**					
rs1001179 C/C	102 (63.0; 55.5–70.4)	61 (64.2; 54.6–73.9)	22 (51.2; 36.2–66.1)	0.95 (0.56–1.61); 0.841; 0.841	1.62 (0.82–3.20); 0.160; 0.645
rs1001179 C/T	55 (34.0; 26.7–41.2)	28 (29.5; 20.3–38.6)	19 (44.2; 29.3–59.0)	1.23 (0.71–2.13); 0.460; 0.704	0.65 (0.33–1.29); 0.215; 0.645
rs1001179 T/T	5 (3.1; 0.4–5.7)	6 (6.3; 1.4–11.2)	2 (4.7; −1.6–10.9)	0.47 (0.14–1.59); 0.218; 0.704	0.65 (0.12–3.49); 0.616; 0.739
rs4880 G/G	34 (21.0; 14.7–27.3)	17 (17.9; 10.2–25.6)	11 (25.6; 12.5–38.6)	1.22 (0.64–2.33); 0.549; 0.704	0.77 (0.35–1.69); 0.519; 0.739
rs4880 G/A	82 (50.6; 42.9–58.3)	54 (56.8; 46.9–66.8)	19 (44.2; 29.3–59.0)	0.78 (0.47–1.30); 0.335; 0.704	1.30 (0.66–2.55); 0.454; 0.739
rs4880 A/A	46 (28.4; 21.5–35.3)	24 (25.3; 16.5–34.0)	13 (30.2; 16.5–44.0)	1.17 (0.66–2.09); 0.587; 0.704	0.92 (0.44–1.91); 0.813; 0.813
**ALLELES**					
rs1001179 C	259 (79.9; 75.6–84.3)	150 (78.9; 73.2–84.7)	63 (73.3; 63.9–82.6)	1.06 (0.68–1.66); 0.788; 0.997	1.46 (0.84–2.85); 0.180; 0.360
rs1001179 T	65 (20.1; 15.7–24.4)	40 (21.1; 15.3–26.8)	23 (26.7; 17.4–36.1)	0.94 (0.61–1.47); 0.788; 0.997	0.69 (0.40–1.19); 0.180; 0.360
rs4880 G	150 (46.3; 40.9–51.7)	88 (46.3; 39.2–53.4)	41 (47.7; 37.1–58.2)	1.00 (0.70–1.43); 0.997; 0.997	0.95 (0.59–1.52); 0.820; 0.820
rs4880 A	174 (53.7; 48.3–59.1)	102 (53.7; 46.6–60.8)	45 (52.3; 41.8–62.9)	1.00 (0.70–1.43); 0.997; 0.997	1.06 (0.66–1.70); 0.820; 0.820

*p*, unadjusted *p* value; Pc, FDR-adjusted *p* value. FDR correction was applied separately to the complete sets of genotype and allele comparisons within each analysis.

## Data Availability

All data relating to the current study, intended for reasonable use, are available from J.A.G. Agúndez (University Institute of Molecular Pathology Biomarkers, University of Extremadura, Cáceres, Spain) and Hortensia Alonso-Navarro and F.J. Jiménez-Jiménez (Section of Neurology, Hospital del Sureste, Arganda del Rey, Madrid, Spain).

## Supplementary Materials

The following supporting information can be downloaded at: https://www.mdpi.com/article/10.3390/biom16101449/s1, The references of previous genetic association studies by our group that included some of the same participants, but analyzed different variants/hypotheses.
