# Supplementary material for: Lack of Association Between CAT rs1001179 and SOD2 rs4880 Variants and Risk for Multiple Sclerosis
Source: Biomolecules. 2026 Oct 4;16(10):1449. doi: 10.3390/biom16101449 (PMC13702394; doi:10.3390/biom16101449)
Supplement: Supplementary file 1 [file biomolecules-16-01449-s001.zip › biomolecules-4540372-supplementary.pdf]

Supplementary materials

Lack of Association Between CAT rs1001179 and SOD2 rs4880 Variants and Risk for Multiple Sclerosis

Elena García-Martín, Javier Gómez-Tabales, José A. G. Agúndez, Julián Benito-León, Jorge Millán-Pascual, María Díaz-Sánchez, Patricia Calleja, Laura Turpín-Fenoll, Hortensia Alonso-Navarro, Esteban García-Albea and Félix Javier Jiménez-Jiménez

There are some genetic association studies conducted by our group in which some of the participants in the present cohort were previously included. Some participants included in the present case-control cohort may have also participated in previous genetic association studies of multiple sclerosis conducted by our research group [1-11]. These earlier studies investigated variants in genes selected according to different biological hypotheses, including histamine metabolism, oxidative stress and detoxification, vitamin D signaling, immune regulation, nitric oxide synthesis, neuronal or myelin-related pathways, and drug-metabolizing enzymes [1-11]. The number of cases and controls varied among these studies because recruitment was conducted over an extended period, eligibility and phenotypic requirements differed between studies, and DNA or complete clinical information was not available for every participant in every analysis. Consequently, the cohorts reported in the earlier publications and the cohort analyzed in the present study are not identical, although partial participant overlap exists. These publications did not analyze the genetic variant or specific hypothesis evaluated in the present study, and no previously generated genotype data were reused as outcome data for the present analysis. The earlier publications are cited to disclose the partial reuse of the underlying clinical cohort and to ensure transparency regarding previous studies involving some of the same participants.

References

1. Agúndez, J. A.; Arroyo, R.; Ledesma, M. C.; Martínez, C.; Ladero, J. M.; de Andrés, C.; Jiménez-Jiménez, F. J.; Molina, J. A.; Alvarez-Cermeño, J. C.; Varela de Seijas, E.; et al. Frequency of CYP2D6 Allelic Variants in Multiple Sclerosis. Acta Neurol. Scand. 1995, 92, 464–467. https://doi.org/10.1111/j.1600-0447.1995.tb09614.x
2. García-Martín, E.; Martínez, C.; Benito-León, J.; Calleja, P.; Díaz-Sánchez, M.; Pisa, D.; Alonso-Navarro, H.; Ayuso-Peralta, L.; Torrecilla, D.; Agúndez, J. A.; Jiménez-Jiménez, F. J. Histamine-N-Methyl Transferase Polymorphism and Risk for Multiple Sclerosis. Eur. J. Neurol. 2010, 17, 335–338. https://doi.org/10.1111/j.1468-1331.2009.02720.x.
3. Martínez, C.; García-Martín, E.; Benito-León, J.; Calleja, P.; Díaz-Sánchez, M.; Pisa, D.; Alonso-Navarro, H.; Ayuso-Peralta, L.; Torrecilla, D.; Agúndez, J. A.; Jiménez-Jiménez, F. J. Paraoxonase 1 Polymorphisms Are Not Related with the Risk for Multiple Sclerosis. Neuromol. Med. 2010, 12, 217–223. https://doi.org/10.1007/s12017-009-8095-9.
4. García-Martín, E.; Lorenzo-Betancor, O.; Martínez, C.; Pastor, P.; Benito-León, J.; Millán-Pascual, J.; Calleja, P.; Díaz-Sánchez, M.; Pisa, D.; Turpín-Fenoll, L.; et al. LINGO1 rs9652490 and rs11856808 Polymorphisms Are Not Associated with Risk for Multiple Sclerosis. BMC Neurol. 2013, 13, 34. https://doi.org/10.1186/1471-2377-13-34.
5. García-Martín, E.; Agúndez, J. A.; Martínez, C.; Benito-León, J.; Millán-Pascual, J.; Calleja, P.; Díaz-Sánchez, M.; Pisa, D.; Turpín- Fenoll, L.; Alonso-Navarro, H.; et al. Vitamin D3 Receptor (VDR) Gene rs2228570 (Fok1) and rs731236 (Taq1) Variants Are Not Associated with the Risk for Multiple Sclerosis: Results of a New Study and a Meta-Analysis. PLoS One 2013, 8, e65487. https://doi.org/10.1371/journal.pone.0065487.

6. Agúndez, J. A.; García-Martín, E.; Martínez, C.; Benito-León, J.; Millán-Pascual, J.; Calleja, P.; Díaz-Sánchez, M.; Pisa, D.; Turpín- Fenoll, L.; Alonso-Navarro, H.; et al. MAPT Gene rs1052553 Variant Is Not Associated with the Risk for Multiple Sclerosis. Hum. Immunol. 2013, 74, 1705–1708. https://doi.org/10.1016/j.humimm.2013.07.008.
7. Agúndez, J. A.; García-Martín, E.; Martínez, C.; Benito-León, J.; Millán-Pascual, J.; Calleja, P.; Díaz-Sánchez, M.; Pisa, D.; Turpín- Fenoll, L.; Alonso-Navarro, H.; et al. NQO1 Gene rs1800566 Variant Is Not Associated with Risk for Multiple Sclerosis. BMC Neurol. 2014, 14, 87. https://doi.org/10.1186/1471-2377-14-87.
8. Agúndez, J. A.; García-Martín, E.; Martínez, C.; Benito-León, J.; Millán-Pascual, J.; Díaz-Sánchez, M.; Calleja, P.; Pisa, D.; Turpín- Fenoll, L.; Alonso-Navarro, H.; et al. The GSTP1 Gene Variant rs1695 Is Not Associated with an Increased Risk of Multiple Sclerosis. Cell. Mol. Immunol. 2015, 12, 777–779. https://doi.org/10.1038/cmi.2014.121.
9. Agúndez, J. A.; García-Martín, E.; Martínez, C.; Benito-León, J.; Millán-Pascual, J.; Díaz-Sánchez, M.; Calleja, P.; Pisa, D.; Turpín- Fenoll, L.; Alonso-Navarro, H.; et al. Heme Oxygenase-1 and 2 Common Genetic Variants and Risk for Multiple Sclerosis. Sci. Rep. 2016, 6, 20830. https://doi.org/10.1038/srep20830. 52
10. Agúndez, J. A. G.; García-Martín, E.; Rodríguez, C.; Benito-León, J.; Millán-Pascual, J.; Díaz-Sánchez, M.; Calleja, P.; Turpín- Fenoll, L.; Alonso-Navarro, H.; García-Albea, E.; et al. Endothelial Nitric Oxide Synthase (NOS3) rs2070744 Polymorphism and Risk for Multiple Sclerosis. J. Neural Transm. 2020, 127, 1167–1175. https://doi.org/10.1007/s00702-020-02211-0.
11. García-Martín, E.; Agúndez, J. A. G.; Gómez-Tabales, J.; Benito-León, J.; Millán-Pascual, J.; Díaz-Sánchez, M.; Calleja, P.; Turpín- Fenoll, L.; Alonso-Navarro, H.; García-Albea, E.; Plaza-Nieto, J. F.; Jiménez-Jiménez, F. J. Association between LAG3/CD4 Genes Variants and Risk for Multiple Sclerosis. Int. J. Mol. Sci. 2022, 23, 15244. https://doi.org/10.3390/ijms232315244
